# Hypoxic targeting and activating TH-302 loaded transcatheter arterial embolization microsphere

**DOI:** 10.1080/10717544.2020.1831102

**Published:** 2020-10-23

**Authors:** Pengkai Ma, Jianhua Chen, Haixian Qu, Ye Li, Xiaohui Li, Xuemei Tang, Zhigang Song, Hainan Xin, Jinbang Zhang, Jingxue Nai, Zhiping Li, Zhijun Wang

**Affiliations:** a School of Chinese Materia Medica, Beijing University of Chinese Medicine, Beijing, China; b Department of Interventional Radiology, The First Medical Center, Chinese PLA General Hospital, Beijing, China; c State Key Laboratory of Toxicology and Medical Countermeasure, Department of Pharmaceutics, Beijing Institute of Pharmacology and Toxicology, Beijing, China; d Department of Pathology, The First Medical Center, Chinese PLA General Hospital, Beijing, China

**Keywords:** Hepatocellular carcinoma, TACE, hypoxia, chemoembolization microsphere, TH-302

## Abstract

The tumor-derived and transcatheter arterial chemoembolization (TACE) induced hypoxia microenvironment is closely related to the poor prognosis of hepatocellular carcinoma (HCC). In this study, hypoxia-activated prodrug TH-302 loaded poly(lactic-co-glycolic acid) (PLGA)-based TACE microspheres were prepared to treat HCC through localized and sustained drug delivery. TH-302 microspheres with three different sizes were fabricated by an oil-in-water emulsion solvent evaporation method and characterized by scanning electron microscopy (SEM), infrared spectra (IR), X-ray diffractometer (XRD), and drug release profiles. The *in vitro* antitumor potential was firstly evaluated in an HepG2 cell model under normoxic and hypoxic conditions. Then, a VX-2 tumor-bearing rabbit model was established and performed TACE to investigate the *in vivo* drug tissue distribution and antitumor efficiency of TH-302 microspheres. Blood routine examination and histopathological examinations were also conducted to evaluate the safety of TH-302 microspheres. TH-302 microspheres with particle size 75–100 μm, 100–200 μm, and 200–300 μm were prepared and characterized by sphere morphology and sustained drug release up to 360 h. Compared with TH-302, the microspheres exhibited higher cytotoxicity, cell apoptosis, and cell cycle S phase retardation in HepG2 cells under hypoxic conditions. The microspheres also displayed continuous drug release in the liver tissue and better anti-tumor efficiency compared with TH-302 injection and lipiodol. Meanwhile, no serious toxicity appeared in the duration of treatment. Therefore, TH-302 microspheres showed to be feasible and effective for TACE and hold promise in the clinical for HCC chemoembolization therapy.

## 1. Introduction

Hypoxia is defined as the concentration of oxygen in tissues less than 2%. Due to the rapid proliferation of tumor cells, incomplete vascular development, and uneven distribution, the supplement of internal oxygen in tumor tissues is insufficient, which eventually leads to the presence of a hypoxic region (Liu et al., 2019). As one of the most important traits of the tumor microenvironment, the vast majority of solid tumors have tumor hypoxia microenvironment, which profoundly influences cellular metabolic reprogramming and further aggravates tumor gene instability and activation of tumor survival factors, causing tumor tolerance to chemotherapy and radiotherapy, and promoting tumor metastasis (Moldogazieva et al., 2020). Given the diverse roles it plays in cancer, hypoxia has emerged as an attractive therapeutic target (Lin & Wu, 2015; Juengpanich et al., 2020).

Hepatocellular carcinoma is one of the highest morbidity and mortality of cancer worldwide. Due to the early symptoms are not obvious, about 70% of patients have been diagnosed at an advanced stage with low surgical resection rate. In these cases, trans-arterial chemoembolization (TACE) has become the standard treatment for Barcelona clinic liver cancer stage B and the subsequent stage (Han & Kim, 2015). In the clinic, conventional TACE (cTACE, represented by Lipiodol^®^ and Gelfoam^®^) and drug-eluting bead TACE (DEB-TACE, represented by DC Bead^®^, Embocept^®^, and HepaSphere^TM^) are common TACE treatment strategies (Kloeckner et al., 2015). However, the non-bioresorbable nature and short-term delivery of chemotherapeutics make these therapeutic strategies unsatisfied clinical outcomes (Li et al., 2015). Even worse, the interruption of blood supply during the chemotherapy process has been found to induce tumor hypoxia more serious. Therefore, there is an urgent need in designing biodegradable, controlled drug release, and tumor hypoxia acting embolization agents for HCC therapy.

Recent endeavors revealed that poly(lactic-*co-*glycolic acid) (PLGA)-based microspheres (MS) could serve as alternative TACE agents due to the high biocompatibility and biodegradability (Han et al., 2016). As a typical example, Occlusin 500^®^, a PLGA/collagen core/shell microsphere with a size of 150–210 µm, exhibited superior embolization efficacy and biocompatibility in a preclinical sheep model. Moreover, the microspheres degraded with time compared with EmboSphere^®^ Microspheres that employed acrylate polymer as the drug carrier (Owen et al., 2012). Choi et al. developed DOX embedded PLGA microspheres, and further trapped hyaluronic acid-ceramide micelle into the microspheres (DOX/HACE MS) to render it with active liver cancer cell targeting ability. The DOX/HACE MS could significantly inhibit liver tumor growth in a McA-RH7777 liver cancer cell implanted rat tumor model (Choi et al., 2015; Lee et al., 2018). Given this, PLGA-based MS hold great promising application as safe biodegradable microspheres for TACE utilization.

TH-302 is a hypoxia-activated prodrug targeting the intra-tumoral hypoxic environment. The dinitroimidazole structure will be fragmented with an alkylating agent dibromoisophosphoramide mustard that selectively binds to the DNA and kills the tumor cells. Thus, it exerts little activity in the normoxic zone and fewer side effects on the normal tissues (Hu et al., 2010). It exhibited superior pre-clinical outcomes when used alone or combined with other chemotherapeutic drugs in several cells and xenograft models, such as non-small cell lung cancer, prostate cancer, melanoma, and pancreatic cancer, etc (Liu et al., 2012). Moreover, encouraging results have been obtained in clinical trials of evofosfamide (TH302) in the treatment of pancreatic cancer, soft tissue sarcoma, and heavily pretreated relapsed refractory multiple myeloma (Phillips, 2016). Therefore, delivering TH-302 to the hypoxia region through PLGA-based TACE MS might be worth exploring for HCC therapy.

**Figure 1.**
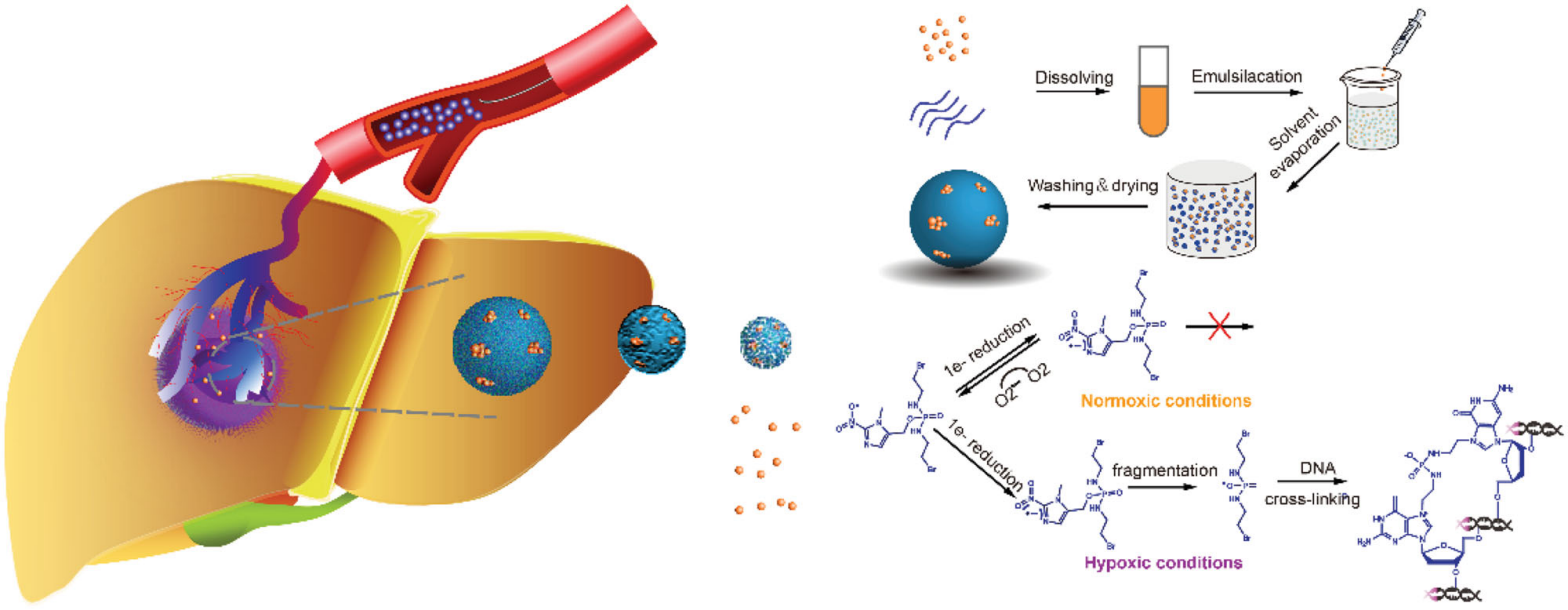
Schematic illustrations of fabrication process of TH-302 MS and its TACE for loco-regionally deliver TH-302 to distal tumor regions to kill tumor cells in the hypoxic microenvironment.

In the study, to overcome drawbacks of cTACE, biodegradable PLGA has been employed to design hypoxia targeting and activating prodrug TH-302 sustain-released TACE MS. As shown in Figure 1, TACE loco-regionally delivers TH-302 MS to distal tumor regions where hypoxic cells reside in. On the one hand, the microspheres exert physical embolism to block the blood supply of tumor tissue, on the other hand, TH-302 prodrugs can be rapidly converted into active components under hypoxic conditions to kill tumor cells. A synergistic anti-tumor effect is obtained to improve the long-term survival rate of patients.

## 2. Materials and methods

### 2.1. Reagents and materials

TH-302 was purchased from Abmole Bioscience Inc (Shanghai, China). PLGA polymers were purchased from Jinan Daigang Biomaterial Co., Ltd (Jinan, Shandong Province, China). PVA-124, methanol, methylene chloride, Tween 20/80, and other chemical reagents were obtained from Sinopharm Chemical Reagent Co., Ltd (Shanghai, China). Dulbecco’s modified Eagle’s medium (DMEM), 3-(4,5-dimethyl-2-thiazolyl)-2,5-diphenyl-2-H-tetrazolium bromide (MTT), and fetal bovine serum (FBS) were purchased from Gibco Life Technology Company (Grand Island, NY). Microguide wire (MAK501N) was purchased from Merit Medical System Inc (South Jordan, UT). Micro-catheter (STD130-22S) was purchased from ASAHI INTECC Co., Ltd. (Tokyo, Japan). A venous indwelling needle (18G) was purchased from B. Braun Medical Inc. (Melsungen, Germany). The contrast agent Ultravist was purchased from Bayer HealthCare Co., Ltd. (Leverkusen, Germany). Hydroxylamine hydrochloride and ketamine hydrochloride were provided by the experimental animal center of the Chinese PLA General Hospital.

### 2.2. Cells and VX-2 tumor-bearing rabbits

VX-2 cells and New Zealand rabbits were provided by the experimental animal center of the Chinese PLA General Hospital. The VX-2 cells revived from being frozen. After cell amplification, the cells were digested and resuspended in saline at a concentration of 5 × 10^7^/mL. To establish VX-2 tumor-bearing passage rabbits, 0.5 mL cell suspension was intramuscular injected into the hind leg of New Zealand rabbits. When the tumor grew to about 2 cm^3^, the tumor was taken off and stored in PBS at 4 °C under aseptic condition. Then, active growing tumor tissues were collected and cut into fragments (1mm^3^). Rabbits were deprivation of fasting and water for 6 h before surgery and anesthetized with 3% pentobarbital sodium solution at a dose of 30 mg/Kg. Following anaesthetization, a 2–3 cm wave was punctured at a location on the left ∼1.5 cm below the xiphoid process, and then the abdominal cavity was opened carefully. Three tumor fragments were implanted into the left medius lobe of the rabbit liver through a puncture needle, staunching with gelatin sponge, and suturing the incision. Rabbits were administrated with penicillin sodium at a dose of 80 wu/d for infection prevention (Mao et al., 2017). All animal experiments were in accordance with Institutional Authority for Laboratory Animal Care.

### 2.3. Fabrication of TH-302 microspheres

An oil-in-water emulsion/solvent evaporation method was modified to prepare TH-302 MS (Wang et al., 2012). Briefly, 0.1 g TH-302 and 0.6 g PLGA were dissolved in methanol/methylene chloride (1/9) mixed solvent and stored at 4 °C for 12 h. The solution was slowly injected into a 15 mL 5% PVA solution using a 2 mL syringe with a 9-gauge needle, homogenized for 5 min to obtain oil-in-water emulsion, and then dispersed in 250 mL 2% PVA solution. The organic solvent was removed under stirring for 5 h at room temperature. The solidified microspheres were collected with a 10 μm sieve and washed three times with 500 mL ultrapure water. Then, wetted microspheres were freezing dried to obtain TH-302 microspheres. The formulation and preparation process, such as PLGA type, molecular weight, terminal type, rotation rate, oil phase injection rate, aqueous pH, and osmotic pressure were optimized based on the entrapment efficiency and particle size. The drug amount was determined by the HPLC method. Microspheres were dissolved by the methanol/methylene chloride (1/9) mixed solvent. Following filtered with a 0.45 μm filter, the drug concentration was detected at 316 nm with a mobile phase of acetonitrile/water (1:1). The encapsulation efficiency (EE) was calculated as the following equation:

EE(%)=(Practical drug loading/theoretical drug loading)×100.

### 2.4. Characterization of TH-302 microspheres

A morphological examination of TH-302 MS was first observed using a light microscope. Then, TH-302 MS were dispersed in water at a concentration of 1 mg/mL, and the particle size was measured with a laser particle size analyzer (Nicomp 380 Zeta Potential/Particle Sizer, Santa Barbara, CA). The lyophilized TH-302 MS was vacuum-coated with platinum and observed using a scanning electron microscopy (SEM) (JSM-840, JEOL, Japan) at an accelerated voltage of 20.0 kV (Ma, Chen, et al., 2018). TH-302, PLGA (9800), physical mixture of TH-302 with PLGA (9800), and TH-302 MS were analyzed by an X-ray diffractometer (D8 Venture, Bruker, Karlsruhe, Germany). The samples were measured with the diffraction angle from 4° to 80°, 2*θ* angle. Additionally, they were also analyzed using the infrared spectra (IR) (Vertex 80, Thermo Fisher, Waltham MA). The scanning range was set from 400 to 4000 cm^−1^.

20 mg microspheres were immersed in 100 mL release medium (pH 7.4, 25 μL Tween 80, 2.5 mL Tween 20) and placed in a constant temperature shaker. The drug release was performed at 100 rpm and 5 mL release medium was withdrawn at predefined intervals. The release medium was centrifuged at 5000 rpm and the supernatant was filtered through a 0.45 μm filter. 1 mL filtrate was collected to determine the drug concentration, the remaining was added to 5 mL and placed back to the release medium. The drug release of TH-302 MS were also performed at pH 6.5 to mimic the drug release profile in the tumor acidic microenvironment. Accelerated drug release was performed at 50 °C and long-term drug release was performed at 37 °C.

### 2.5. Cytotoxicity

MTT assay method was used to evaluate the cytotoxicity of TH-302 and TH-302 MS under hypoxic or normoxic conditions. Briefly, 100 μL HepG2 cells were seeded in 96 well plates at a concentration of 5 × 10^3^ cells/well. After reaching the logarithmic growth phase, 100 μL drug solutions at different concentrations (0.78–100 μM) were added in the wells, and each concentration was performed in six replicates. For hypoxic groups, the 96-well plates were placed in the AnaeroPack for 24 h or 48 h at 37 °C (Sun et al., 2014). For normoxic groups, the 96-well plates were directly placed in the cell incubator for 24 h or 48 h. Afterward, 20 μL MTT at a concentration of 5 mg/mL was added in each well and incubated for another 4 h. Finally, the culture medium was withdrawn and 150 μL DMSO was added to dissolve the formazan crystal. The absorbance at 490 nm of each well was recorded using a microplate reader (Varioskan LUX, Thermo Fisher).

### 2.6. Cell apoptosis

Annexin V-FITC/PI staining method was used to evaluate the cell apoptosis (Ma, Sun, et al., 2018). Briefly, 3 mL HepG2 cells were seeded in 6 well plates at a concentration of 1.6 × 10^5^ cells/well. After reaching the logarithmic growth phase, 3 mL drug solutions at different concentrations were added in the wells, and each concentration was performed at six replicates. For hypoxic groups, the 6-well plates were placed in the AnaeroPack for 24 h at 37 °C. For normoxic groups, the 96-well plates were directly placed in the cell incubator for 24 h. Following the culture medium was withdrawn, cells were trypsinized without EDTA, collected at 1800 rpm/min for 5 min, and washed with pre-cooling PBS three times. Afterward, 5 μL YF488-Annexin V and 5 μL PI working solution was added in each well and co-incubated with cells for 15 min. Cell apoptosis was determined using a flow cytometer (CytoFLEX LX, Beckman Coulter, Pasadena, CA).

### 2.7. Cell cycle

PI staining method was used to evaluate the cell cycle (Li et al., 2016). The cell culture and drug dosing were the same with cell apoptosis evaluation. Cells were trypsinized, collected at 1800 rpm/min for 5 min, washed with pre-cold PBS three times, and fixed with 1 mL 4 °C pre-cooling 75% ethanol overnight at −20 °C. Following removing the ethanol, 0.5 mL PI working solution was added in each well and co-incubated with cells for 15 min. Then, the cell cycle was determined using a flow cytometer (CytoFLEX LX, Beckman Coulter).

### 2.8. Hepatic artery angiography and embolization

Transcatheter super-selective hepatic artery embolization was conducted for administrating TH-302 MS. Rabbits were deprivation of fasting and water for 8 h before surgery. Following anesthesia with xylazine hydrochloride injection and ketamine hydrochloride injection (V/V, 1:1) at a dose of 0.3 mL/kg, the femoral artery was bluntly isolated. Replace the femoral artery sheath with an 18G venous indwelling needle and penetrate the femoral artery. After the needle and the hose entered the artery and the blood returned to the hose, the needle was withdrawn into the soft sheath. Then the soft sheath was slowly pushed forward into the blood vessel by about 1 cm. Fully pull out the needle, connect the three-way connector to stop bleeding. 1 mL contrast agent was injected to confirm that the femoral artery sheath was all located in the artery. Introducing a 2.2 F microcatheter via the three-way connector, the microwire guides the super-selection into the proper hepatic artery. Digital subtraction angiography (DSA) was used to define the anatomical details of the hepatic artery and branching, micro-guidewire guided further super-selective insertion into the left hepatic artery opening. Hepatic enhancement CT (Somatom Emotion 16, Siemens) was used to monitor the tumor size. Following anaesthetization, rabbits were administrated with contrast media Ultravist (370 mgI/mL) through indwelling 18G saving needles in-ear vein (pressure 200 KPa, dosage 6 mL, injection rate 1.0 mL/s). Plain and dual-phase contrast-enhanced CT scanning were performed to observe the tumor with a scan delay of 20 s (Zhang et al., 2014).

### 2.9. Tissue distribution

Seventy-five rabbits were randomly divided into 5 groups, which were TH-302 MS_75–100_, TH-302 MS_100–200_, TH-302 MS_200–300_, TH-302 lipiodol (IOD), and TH-302 injection, respectively. The rabbits were sacrificed by CO_2_ at 24 h, 48 h, 72 h, 5 d, 7 d, post-injection. The liver, kidney, and heart tissues were collected and weighted. Approximately 50 g tissues were cut into 1 × 1 × 1 cm^3^ fragments and homogenized with saline. The supernatant was collected with centrifuging at 10,000 rpm for 5 min and then precipitated with 5 mL methanol. The methanol was evaporated under a stream of nitrogen and re-dissolved in 0.5 mL methanol, then the TH-302 concentration was determined using the HPLC method.

### 2.10. Antitumor efficacy

Embolization was performed when the maximum length of the tumor reached 1.5 cm. The maximum length (a) and the width (b) of tumors were measured, and rabbits with maximum tumor length between 1.5 cm and 2.0 cm were in accordance with the including criteria for study. The tumor volume was recorded at 3 d, 7 d, and 14 d post-embolization and calculated with the formulation: *V* = *ab*^2^/2. On day 14, the rabbits were sacrificed by carbon dioxide asphyxiation. The livers were collected and cut the tumors off the livers. The tumors were fixed with 10% paraformaldehyde for 48 h and cut into 5 mm sections for H&E staining. Anti-tumor efficacy was evaluated by the modified Response Evaluation Criteria in Solid Tumors (mRECIST) (Zuckerman et al., 2014).

### 2.11. Safety

Peripheral venous blood was collected at pre-embolization and 3 d, 5 d, 7 d post-embolization. Routine biochemistry assays were conducted to detect the physiological-biochemical indexes, such as ALT, AST, CREA, and UREA. Moreover, normal tissues such as liver, kidney, spleen, heart, and lung were collected and processed for H&E staining. These indexes were used to evaluate the potential toxicity of TH-302 MS to normal tissues.

### 2.12. Statistical analysis

All data were reported as mean ± standard deviation (SD). Statistical significant differences between groups were analyzed using *t*-tests and analysis of variance. The *t*-test was used when the variance between groups was not uniform or did not obey the normal distribution. A value of *p* < .05 was considered statistically significant.

## 3. Results and discussion

### 3.1. Fabrication of TH-302 microspheres

**Figure 2.**
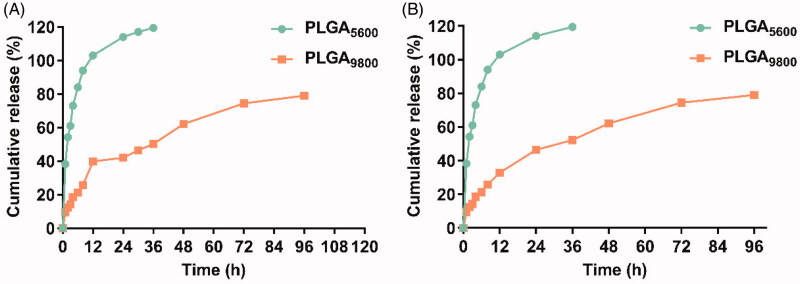
Drug release profiles from TH-302 MS prepared from PLGA with different molecular weight. (A) Accelerated drug release at 50 °C; (B) Long-term drug release at 37 °C.

The key factors influencing the quality of TH-302 MS were investigated to optimize the preparation process. First, the PLGA type (LA/GA molar ratio), molecular weight, and terminal type of PLGA were optimized with the entrapment efficiency (EE) of TH-302 MS as evaluation indexes. As shown in Table 1, the LA/GA molar ratio of PLGA had a negligible influence on EE and the higher proportion of lactic acid the higher EE of TH-302 MS. The molecular weight and terminal groups of PLGA hold a big contribution toward the EE. The microspheres prepared from PLGA terminated with the ester group had the highest EE. And, the microspheres prepared from PLGA 5600 and PLGA 9800 had higher EE compared with PLGA 22,000 and 33,000. As shown in Figure 2, TH-302 MS prepared from different molecular weight PLGA showed different accelerated drug release profiles. TH-302 MS (PLGA 5600) released 90% drug within 12 h, while TH-302 MS (PLGA 9800) released 40% in the same duration. As for long-term drug release, TH-302 MS (PLGA 5600) was released completely within 36 h, while TH-302 MS (PLGA9800) released no more than 80% within 96 h. These results indicated that the microspheres prepared from PLGA 9800 showed less burst release and a more sustained drug release profile. The pH and osmotic pressure of the aqueous phase had no significant influence on the EE of microspheres. Therefore, the ester-terminated PLGA (75/25, 9800) was finally selected for the preparation of TH-302 MS.

**Table 1.** Influence of PLGA on the EE, DL, and particle size of TH-302 MS.

Formulation variables							Parameters
PLGA type (molar ratio)	Molecular weight	Terminal type	Rotation rate	Injection rate	pH	Osmotic pressure	EE (%)
PLGA (80/20)	5600	Ester	350 rpm	1 mL/75s	7.0	5% PVA	34.78
PLGA (75/25)	5600	Ester	350 rpm	1 mL/75s	7.0	5% PVA	33.42
PLGA (50/50)	5600	Ester	350 rpm	1 mL/75s	7.0	5% PVA	32.19
PLGA (75/25)	5600	Ester	350 rpm	1 mL/15s	7.0	5% PVA	56.53
PLGA (75/25)	5600	Carboxyl	350 rpm	1 mL/15s	7.0	5% PVA	54.67
PLGA (75/25)	5600	Hydroxyl	350 rpm	1 mL/15s	7.0	5% PVA	39.47
PLGA (75/25)	5600	Ester	350 rpm	1 mL/15s	5.0	5% PVA	56.00
PLGA (75/25)	5600	Ester	350 rpm	1 mL/15s	9.2	5% PVA	57.36
PLGA (75/25)	5600	Ester	350 rpm	1 mL/15s	7.0	5% PVA +11.5% NaCl	58.17
PLGA (75/25)	9800	Ester	350 rpm	1 mL/15s	7.0	5% PVA	46.00
PLGA (75/25)	22,000	Ester	350 rpm	1 mL/15s	7.0	5% PVA	18.60
PLGA (75/25)	33,000	Ester	350 rpm	1 mL/15s	7.0	5% PVA	18.60

Then, the rotation rate and injection rate were optimized with a particle size as evaluation indexes. As shown in Table 2, the particle size increased with the decrement of rotation rate. This could be explained that faster rotation speed produced a larger shearing force, which produced smaller oil droplets. Likewise, the faster the oil phase was injected into the aqueous phase, the greater the shear force and the smaller the diameter of the microspheres. Finally, microspheres in three sizes (75–100 μm, 100–200 μm, and 200–300 μm) were prepared through adjusting the injection rate.

**Table 2.** Influence of rotation rate and injection rate on the particle size of TH-302 MS.

Formulation variables							Parameters
PLGA type (molar ratio)	Molecular weight	Terminal type	Rotation rate	Injection rate	pH	Osmotic pressure	Particle size (μm)
PLGA (75/25)	9800	Ester	350 rpm	1 mL/9s	7.0	5% PVA	119
PLGA (75/25)	9800	Ester	400 rpm	1 mL/9s	7.0	5% PVA	87
PLGA (75/25)	9800	Ester	600 rpm	1 mL/9s	7.0	5% PVA	75
PLGA (75/25)	9800	Ester	350 rpm	1 mL/15s	7.0	5% PVA	270
PLGA (75/25)	9800	Ester	350 rpm	1 mL/25s	7.0	5% PVA	195
PLGA (75/25)	9800	Ester	350 rpm	1 mL/75s	7.0	5% PVA	88

### 3.2. Characterization of TH-302 microspheres

**Figure 3.**
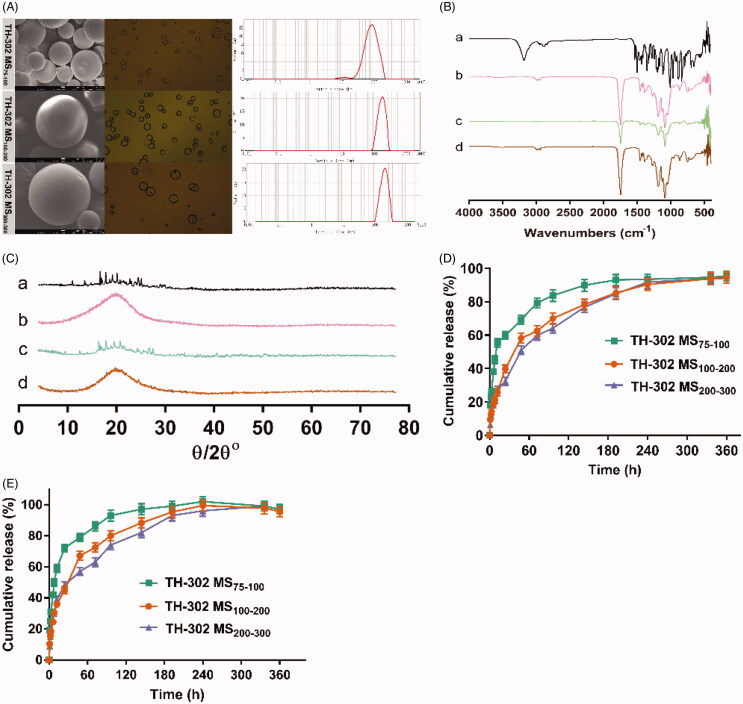
Characterization of TH-302 MS. (A) Morphology and particle size of different sized TH-302 MS; (B) IR spectra of (a) TH-302, (b) PLGA, (c) physical mixture of TH-302 and PLGA, and (d) TH-302 MS; (C) XRD profiles of (a) TH-302, (b) PLGA, (c) physical mixture of TH-302 and PLGA, and (d) TH-302 MS; (D) *In vitro* drug release profile of different sized TH-302 MS (pH 7.4); (E) *In vitro* drug release profile of different sized TH-302 MS (pH 6.5).

In the study, TH-302 MS with different sizes were prepared. As shown in Figure 3(A), the morphology of TH-302 MS was spherical in shape with a smooth and nonporous surface. The microspheres were not adhered to each other, which indicated their good dispersity. The particle size of TH-302 MS was 75–100 μm (average particle size 99.46 μm), 100–200 μm (average particle size 198.60 μm), and 200–300 μm (average particle size 272.08 μm), respectively. As shown in Figure 3(B), TH-302 shared characteristic absorption peaks at 3190 and 2880 cm^−1^. PLGA shared characteristic absorption peaks at 1747 cm^−1^, which still existed in the physical mixture of TH-302 with blank microspheres and TH-302 MS. As XRD showed in Figure 3(C), TH-302 exhibited crystal diffraction peaks in the range of 10°−30°, which still presented in the physical mixture except for the intensity decreased. It indicated that TH-302 was in the crystalline form. However, the characteristic peaks for TH-302 disappeared in TH-302 MS. The results of IR and XRD demonstrated that TH-302 was encapsulated in the microspheres.

The *in vitro* drug release was performed to estimate the *in vivo* drug release behavior. As shown in Figure 3(D), TH-302 MS with different particle sizes showed different release profiles *in vitro*. Overall, all TH-302 MS possessed biphasic release pattern during the 15-d period at pH 7.4. There was an initial burst release in the first phase and then a sustained release in the second phase. TH-302 MS (75–100 μm) released 69.20% compared with 58.12% and 50.96% for TH-302 MS (100–200 μm) and TH-302 MS (200–300 μm) during 48 h, which indicated that the initial burst release decreased with the increase of particle size. Moreover, TH-302 MS (100–200 μm) and TH-302 MS (200–300 μm) exhibited more sustained drug release than TH-302 MS (75–100 μm). Therefore, it could be inferred that the drug release profile of TH-302 MS was related to their particle size. On the one side, the smaller MS had a larger specific surface area and adhered more drug on the surface, which caused more serious drug burst release. On the other side, the release medium infiltrated into the interior of smaller MS easier, so the drug diffused into the release medium easier. Moreover, as shown in Figure 3(E), all TH-302 MS exhibited faster drug release at pH 6.5 than at pH 7.4, they released completely within 10 d. TH-302 MS (100–200 μm) and TH-302 MS (200–300 μm) also exhibited more sustained drug release than TH-302 MS (75–100 μm) at tumor acidic microenvironment. Therefore, the drug release profiles of TH-302 MS (100–200 μm) and TH-302 MS (200–300 μm) might be more suitable for TACE therapy.

### 3.3. Cytotoxicity

**Figure 4.**
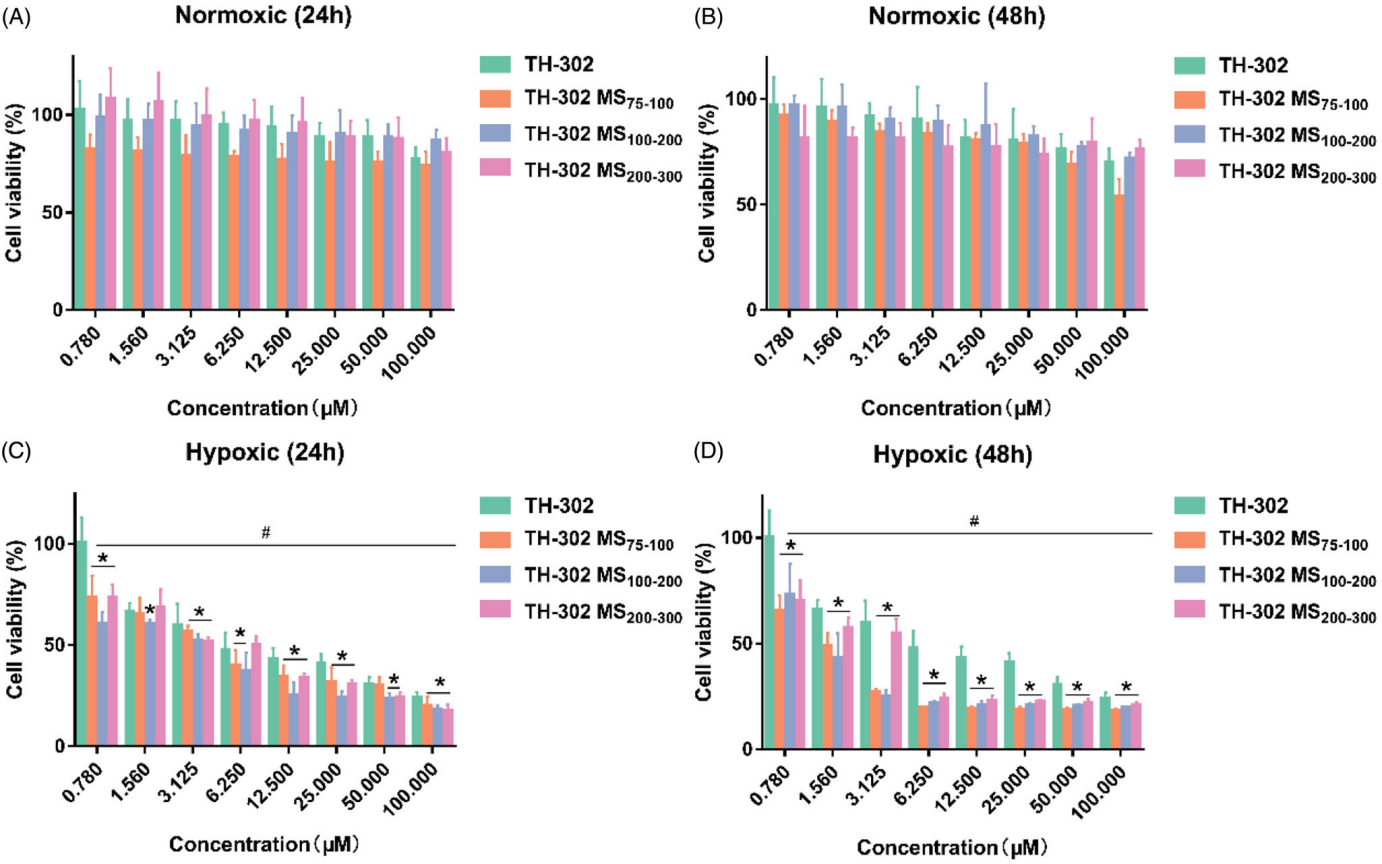
Cytotoxicity of TH-302 and TH-302 MS on the HepG2 cell under normoxic (A, 24 h; B, 48 h) or hypoxic conditions (C, 24 h; D, 48 h). *p < .05, compared with TH-302 under hypoxic condition; ^#^*p* < .05 compared with the corresponding groups under normoxic condition.

The *In vitro* anti-tumor potential of the TH-302 MS was firstly assessed by cytotoxicity assays in HepG2 cells. As shown in Figure 4, the cytotoxicity of TH-302 and its microspheres were concentration-dependent under both normoxic and hypoxic conditions. And the cytotoxicity under hypoxic conditions was significantly higher than normoxic conditions, which demonstrated that the TH-302 could be hypoxia-activated. It also could be predicted that the TH-302 and its microspheres would be less toxic to normal tissues. The cytotoxicity of TH-302 microspheres was significantly higher than free drug TH-302 under hypoxic condition. Moreover, different sized microspheres showed different cytotoxicity under both normoxic and hypoxic conditions. At 24 h, the IC50 of TH-302, TH-302 MS (75–100 μm), TH-302 MS (100–200 μm), and TH-302 MS (200–300 μm) were 6.48 μM, 2.77 μM, 5.18 μM, and 3.19 μM, respectively, the TH-302 MS (75–100 μm) showed lower cytotoxicity than other microspheres at any concentrations. At 48 h, the IC50 of them were 6.25 μM, 1.78 μM, 1.19 μM, and 3.89 μM, respectively. The IC50 of TH-302 MS was significantly lower than the TH-302 drug (*p* < .05), which demonstrated that the TH-302 MS had stronger cytotoxicity than the TH-302 drug. Interestingly, TH-302 MS (75–100 μm) showed higher cytotoxicity than other microspheres when the drug concentration was higher than 6.25 μM. This phenomenon might be related to the different drug release profiles of different sized microspheres, and the released TH-302 need time to be activated.

### 3.4. Cell apoptosis

**Figure 5.**
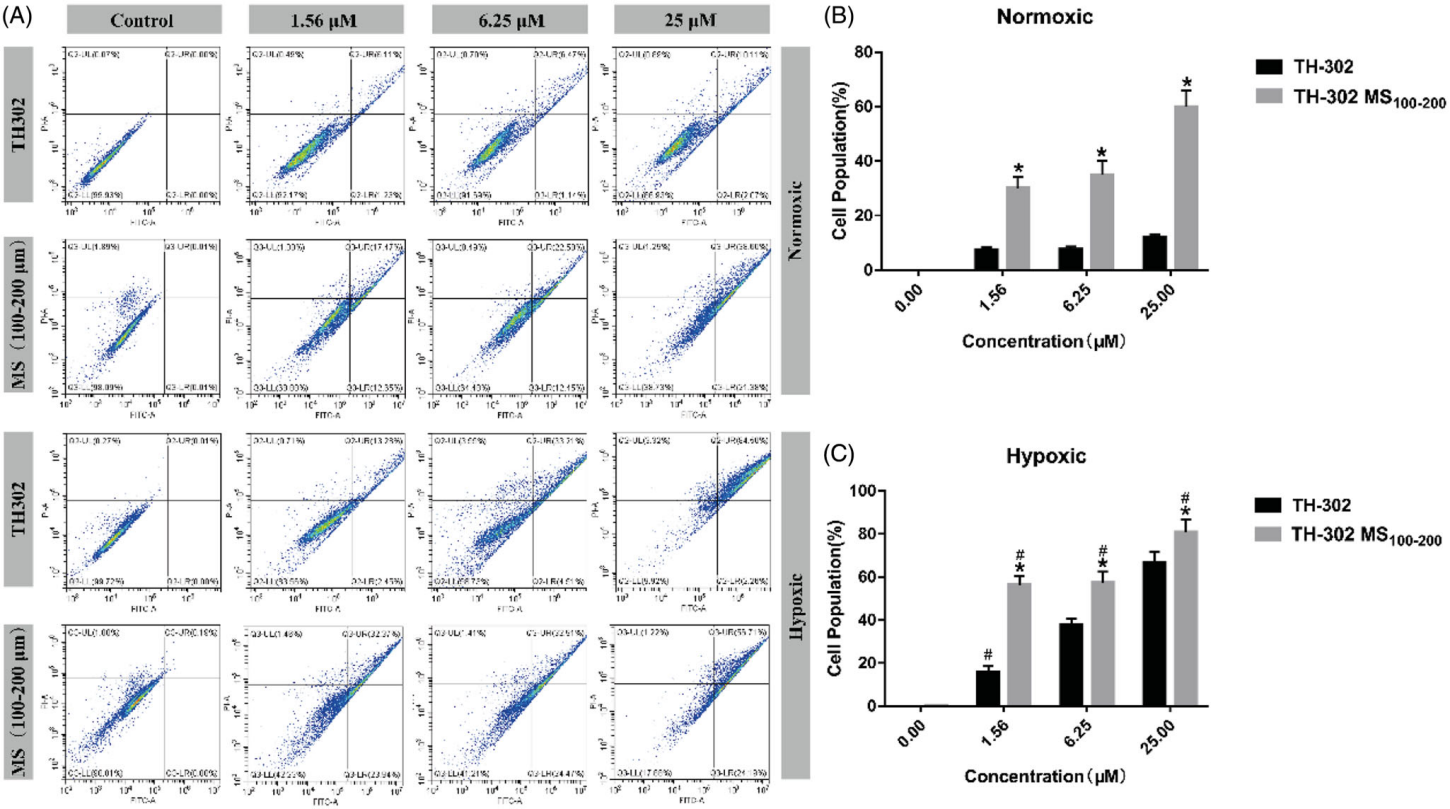
Apoptotic efficacy of TH-302 and TH-302 MS (100–200 μm). (A) Apoptosis distribution of HepG2 cells post-treatment with TH-302 and TH-302 MS (100–200 μm) under normoxic and hypoxic conditions; (B) Apoptotic cell population under normoxic condition; (C) Apoptotic cell population under hypoxic condition. *p < .05, compared with TH-302.

The *In vitro* anti-tumor potential of the TH-302 MS was then assessed by apoptosis assays in HepG2 cells. As shown in Figure 5, for the negative control group (without drug), the cell apoptosis had no significant difference under normoxic or hypoxic conditions (*p* > .05). Both TH-302 and TH-302 MS could concentration-dependently induce cell apoptosis, and TH-302 MS showed a significantly higher apoptotic rate than TH-302 under hypoxic condition or normoxic condition at the same concentration (*p* < .05). Moreover, TH-302 and TH-302 MS had a higher apoptotic rate under hypoxic conditions than normoxic conditions, which further demonstrated that the TH-302 could be activated by hypoxia and TH-302 MS could enhance the inducing apoptosis effect of TH-302. Interestingly, TH-302 MS, unlike TH-302 free drug, also exhibited a pro-apoptotic effect under normoxic conditions. It might be explained that due to the large particle size and weight of TH-302 MS (100–200 μm), they easily sank onto the cells and disturbed their regular growth.

### 3.5. Cell cycle

**Figure 6.**
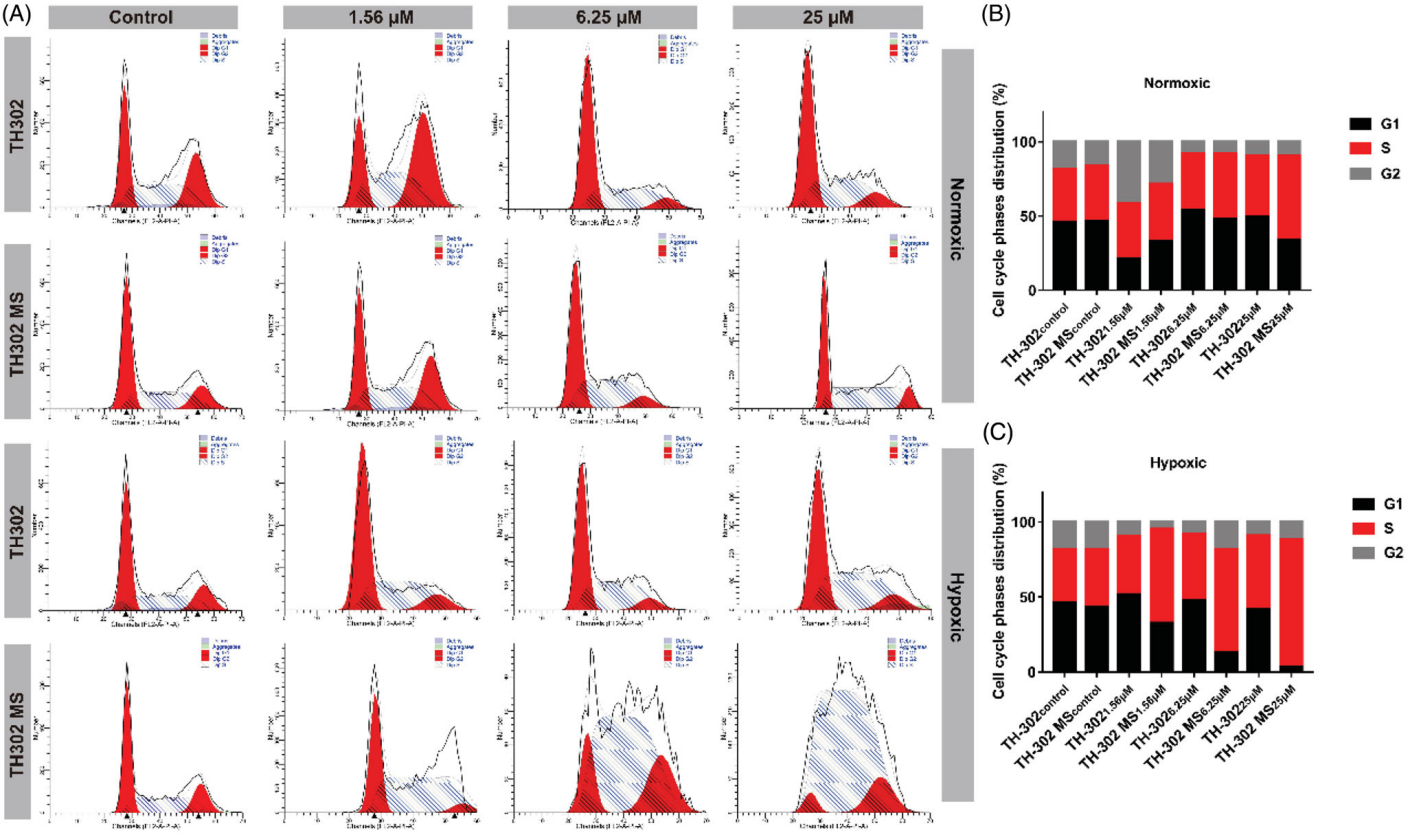
(A) Cell cycle phase distribution of HepG2 post-treatment with TH-302 and TH-302 MS (100–200 μm) under normoxic and hypoxic conditions; (B) Cell cycle S phase distribution of HepG2 post-treatment with TH-302 and TH-302 MS (100–200 μm) under normoxic condition; (C) Cell cycle S phase distribution of HepG2 post-treatment with TH-302 and TH-302 MS (100–200 μm) under hypoxic condition.

The effects of TH-302 and TH-302 MS (100–200 μm) on the regulation of the cell cycle was also investigated. As shown in Figure 6, under normoxic condition, the S phase distribution ratio for TH-302 at a concentration of 0, 1.56, 6.52, and 25 μM were 35.31, 37.21, 38.08, and 40.68%, respectively. As for TH-302 MS (100–200 μm), the S phase distribution ratio was 35.34, 38.68, 49.04, and 56.47%, respectively. Therefore, TH-302 and TH-302 MS (100–200 μm) exhibited S cell cycle arrest at high drug concentration (6.52 or 25 μM) under normoxic conditions. While, under hypoxic conditions, the S phase distribution of TH-302 at concentrations of 6.52, and 25 μM increased to 44.28 and 49.04%, respectively. As for TH-302 MS (100–200 μm), the S phase distribution at concentrations of 1.56, 6.52, and 25 μM increased to 62.62, 68.75, and 85.39%, respectively. Therefore, TH-302 and TH-302 MS (100–200 μm) exhibited stronger cell cycle arrest ability under hypoxic conditions compared with the normoxic conditions, and TH-302 MS (100–200 μm) could significantly enhance the cell cycle arrest ability of TH-302. These results demonstrated that TH-302 MS (100–200 μm) could inhibit the proliferation of HepG2 cells by arresting the cell cycle.

### 3.6. Tissue distribution

**Figure 7.**
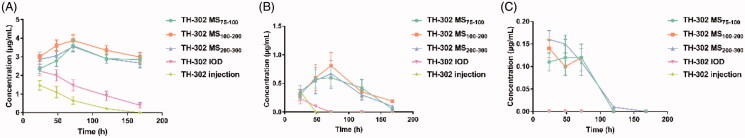
TH-302 concentration in (A) liver, (B) kidney and (C) heart tissues following TACE using TH-302 MS, TH-302 IOD, and TH-302 injection in the rabbits (*n* = 3).

The drug distribution in the liver, kidney, and heart tissues after TACE was shown in Figure 7. The TH-302 concentration of TH-302 MS in these tissues was significantly higher than TH-302 IOD and TH-302 injection, and the TH-302 concentration of TH-302 MS in the liver was significantly higher than the heart and kidney. It reached the maximum concentration at 72 h following TACE and then decreased gradually with time extending and the TH-302 concentration was still above 2.5 μg/mL until 168 h. Whereas the TH-302 IOD and TH-302 injection showed rapid elimination characterization in heart and kidney tissues, the drug concentration in heart and kidney was almost could not be detected after 24 or 72 h. These results demonstrated that the TH-302 MS possessed sustained drug release profiles *in vivo*. Moreover, three different sized TH-302 MS exhibited similar drug tissue distribution, the drug concentration of TH-302 (100–200 μm) in the liver was slightly higher than TH-302 (75–100 μm) and TH-302 (200–300 μm). Though the drug concentration of TH-302 MS in the heart and kidney were higher than TH-302 IOD and TH-302 injection, it would not induce severe toxicity to these tissues because TH-302 would not be activated in normal tissues.

### 3.7. Antitumor efficacy

**Figure 8.**
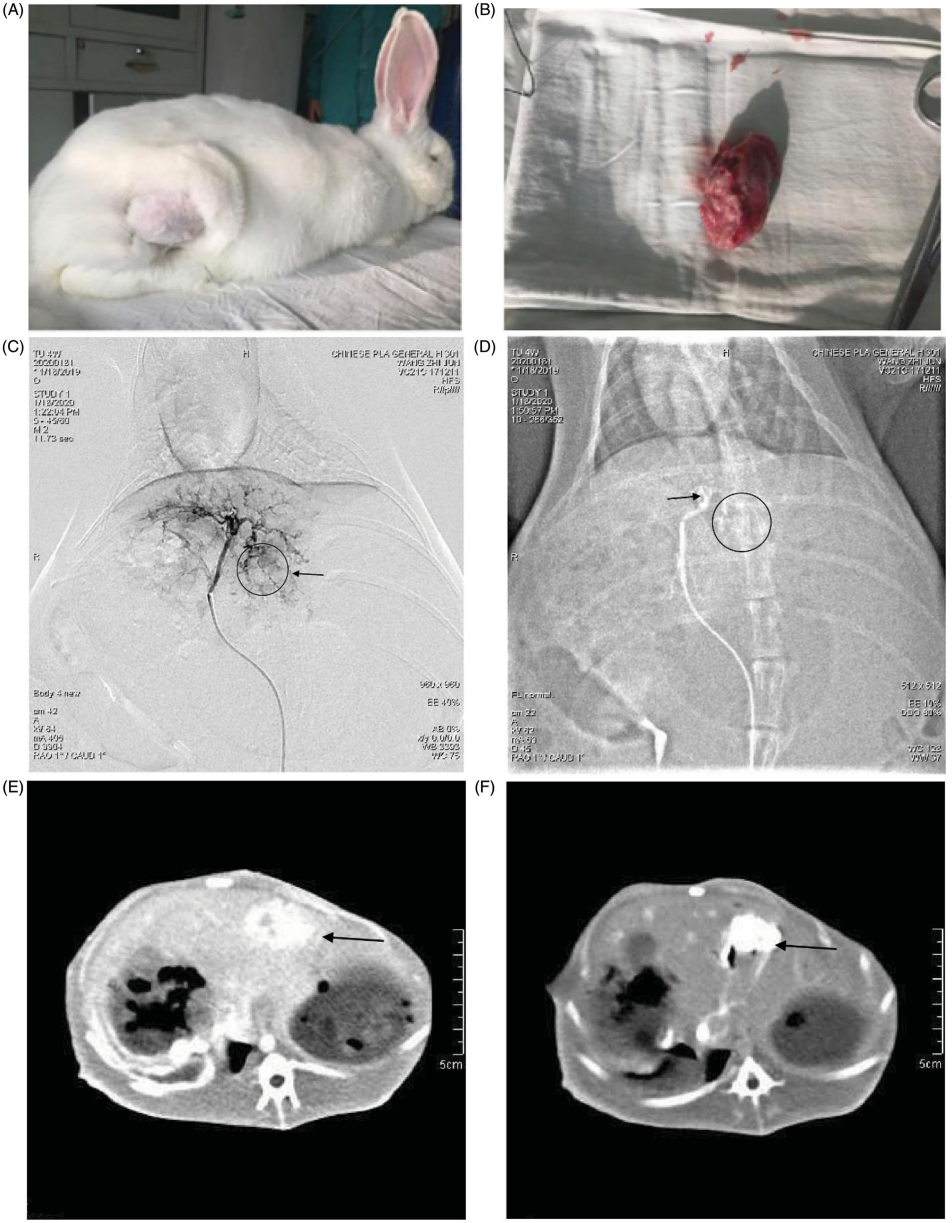
(A, B) VX-2 tumor bearing passage rabbits and its implanted tumor;(C, D) arterial angiogram of VX-2 tumor bearing rabbits before and immediately after embolization; (E, F) hepatic enhancement CT before and immediately after embolization.

VX-2 tumor-bearing rabbits model was first established to evaluate the anti-tumor efficacy of TH-302 MS. As shown in Figure 8(A,B), the VX-2 tumor was successfully implanted in the leg of the passage rabbit to provide sufficient VX-2 tumor cells. In Figure 8(C,D), angiographic photos for the liver of the rabbits before and after embolization showed that blood flow was obstructed following TH-302 MS and was injected into the liver artery. The tumor was also observed through hepatic enhancement CT examination, as shown in Figure 8(E,F), lamellar CT scan for the tumor displayed hypodensity in the center and a dynamic CT Scan displayed boundary enhancement of the tumor. These results proved the embolization of rabbit liver artery with TH-302 MS was successfully achieved in this experiment.

**Figure 9.**
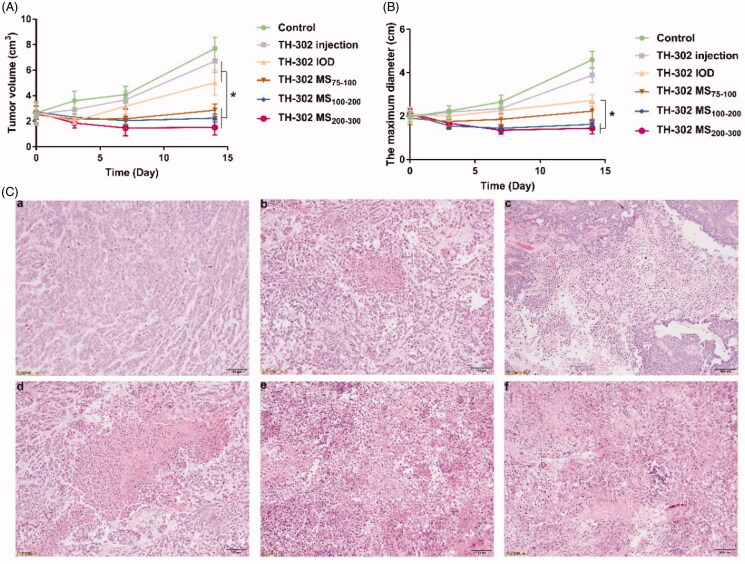
Antitumor activity of TH-302 MS, TH-302 injection and TH-302 IOD in VX-2 tumor bearing rabbits. (A) Tumor volume-time curves for each group following TACE within 14 d; (B) tumor diameter–time curves for each group following TACE within 14 d; (C) histopathological examinations for liver tumors. Data are expressed as the means ± SD (*n* = 6). *p < .05, compared with control; ^#^*p* < .05 compared with TH-302 IOD.

The *in vivo* anti-tumor efficacy was further investigated in the VX-2 tumor-bearing rabbit model. As shown in Figure 9(A,B), there was no significant difference in the baseline value of preoperative tumor volume between the groups (*p >* .05). Rabbits treated with different sized TH-302 microspheres or TH-302 IOD showed obvious retardation of tumor growth compared with the control group. Interestingly, the tumor volume of all treated groups decreased during the first 3 d. However, the tumor volume of the TH-302 IOD group increased quickly in the following 4 d. Most importantly, treatment with TH-302 microspheres exhibited a more remarkable slow-down for tumor growth relative to the TH-302 IOD group (*p* < .05). Meanwhile, different sized TH-302 microspheres showed varying anti-tumor efficacy, and the anti-tumor efficacy increased with the particle size of microspheres. This phenomenon might be caused by the better embolization efficacy and more sustained drug release of microspheres with bigger particle size. Moreover, H&E staining of the tumor (Figure 9(C)) showed that the control group had higher tumor cell density than other groups, which indicated its high malignant degree. And the administration groups exhibited therapeutic effects with different degrees. There were small necrotic foci in the TH-302 injection and TH-302 IOD groups, while large necrosis area could be found in the TH-302 MS groups. These results further demonstrated TH-302 MS had better therapeutic effects than TH-302 injection and TH-302 IOD.

According to the sum of the maximum diameters of target lesions, the short-term anti-tumor efficacy was evaluated by the modified response evaluation criteria in solid tumor (mRECIST). As shown in Table 3, taking the smallest sum of the maximum diameters of target lesions as a reference, the increase of the sum of the maximum diameters of target lesions for control, TH-302 injection, and TH-302 IOD groups were 76.36 and 18.78. And new lesions appeared during the treatment. Therefore, the TH-302 injection and TH-302 IOD were evaluated as PD, which indicated that the efficacy of TH-302 was not sufficient when iodized oil was used as a carrier. As for TH-302 MS_75–100_ group, the increase of the sum of the maximum diameters of target lesions was 5.19% and no new lesion appeared, so it was evaluated as SD. While, for TH-302 MS_75–100_ and TH-302 MS_200–300_ groups, the decrease of the sum of the maximum diameters of target lesions were 31.06 and 32.40%, and no new lesions appeared. The TH-302 MS_100–200_ and TH-302 MS_200–300_ exhibited superior anti-tumor potential, they were evaluated as PR.

**Table 3.** Short-term anti-tumor efficacy evaluated by the mRECIST.

	Target lesions	Non-target lesions	New lesions	Overall efficacy
Control	PD	PD	Yes	PD
TH-302 injection	PD	PD	Yes	PD
TH-302 IOD	SD	PD	Yes	PD
TH-302 MS_75–100_	SD	NON-PD	No	SD
TH-302 MS_100–200_	PR	NON-PD	No	PR
TH-302 MS_200–300_	PR	NON-PD	No	PR

*Note*. CR: complete response, disappearance of all lesions; PR: partial response, at least a 30% decrease in the sum of diameters of target lesions, taking as reference the baseline sum diameters; PD: progressive disease, at least a 20% increase in the sum of diameters of target lesions, taking as reference the smallest sum on study; SD: stable disease, between PR and PD; NON-PD: persistence of one or more non-target lesions.

### 3.8. Safety

**Figure 10.**
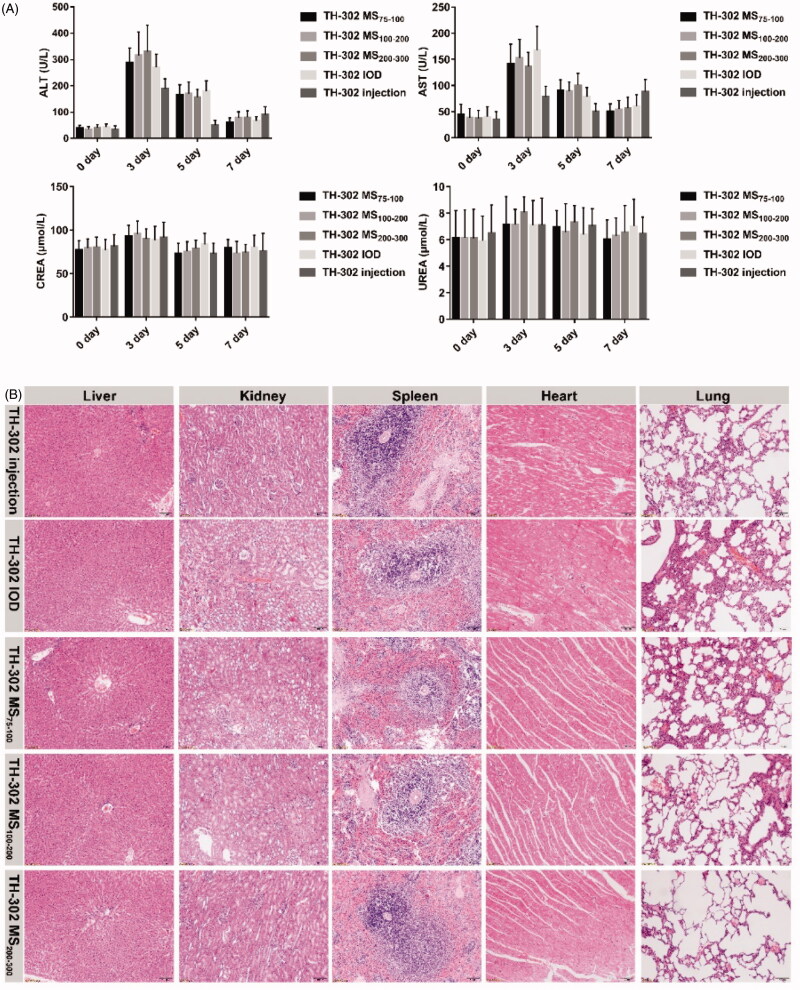
Evaluation *in vivo* toxicity of TH-302 MS, TH-302 injection and TH-302 IOD in VX-2 tumor bearing rabbits. (A) The serum levels of ALT, AST, CREA and UREA following TACE within 14 d; (B) histopathological examinations for normal tissues after TACE. Data are expressed as the means ± SD (*n* = 6). *p < .05.

All animals survived until 14 d after surgery with no serious complications. As shown in Figure 10(A), the ALT and AST values of each group significantly increased 3 d after embolization (*p* < .05). The main reason was due to the embolization effect of microspheres and the side effects of contrast agents. There was a progressive decline 5 d after surgery. At 7 d post-embolization, the ALT and AST values reduced to the level pre-embolization, indicating that liver function could recover 7 d after surgery. TH-302 MS and TH-302 IOD showed comparable ALT and AST values, while TH-302 injection showed slightly less ALT and AST values, which might be due to the lower drug concentration in the liver. CREA and UREA were slightly elevated in all groups 3 d after embolization. There were no significant differences among different groups, indicating that TH-302 MS had no serious kidney toxicity. And the H&E staining results showed that the histological morphologies of the liver, kidney, spleen, heart, and lung were normal, which demonstrated there were no serious pathological changes.

## 4. Conclusions

TH-302 MS with particle size 75–100 μm, 100–200 μm, and 200–300 μm were successfully prepared by the O/W method for TACE treatment of HCC. The fabricated TH-302 MS provided sustained drug release for more than 14 d *in vitro* and *in vivo* after TACE. More interestingly, the TH-302 MS demonstrated enhanced cytotoxicity, cell apoptosis, and cell cycle S phase arrest on HepG2 cells under hypoxic conditions. In the VX-2 tumor-bearing rabbits model, the intra-arterial administration of TH-302 MS exhibited a sufficient embolic effect, localized drug distribution, and low systemic drug exposure. Moreover, the antitumor efficacy of TH-302 MS was significantly superior to the TH-302 IOD or TH-302 injection. In summary, the developed TH-302 MS provided an efficient candidate for TACE treatment of HCC.
